# The Treatment of Abscesses of the Neck

**Published:** 1884-02

**Authors:** 


### The Treatment of Abscesses of the Neck.

Dr. John A. Lidell, in a very instructive article on this subject in a number of *The American Journal of the Medical Sciences*, points out that sudden death may occur from deep-seated abscesses of the neck, or the continuance of life may be greatly endangered, much oftener than is generally supposed; and that these abscesses in the neck are more frequently attended with hemorrhages due to the opening of important blood vessels by ulceration or erosion, and by *ramollissement* consequent upon the disorders themselves, than abscesses in the other surgical regions. The superior liability of cervical abscesses to the spontaneous occurrence of dangerous hemorrhages arises in part from the greater number and importance of the cervical blood vessels; but more particularly from the inanition and exhaustion, or low state of the constitutional powers, and consequent feebleness of the reparative forces, which rapidly result from most of the deep abscesses of the neck, or rather from the inability to swallow enough food to support life, and from the powerlessness to get any refreshing sleep, or even repose, with which these abscesses are oftentimes attended. The septic or toxæmic influence of the fetid secretions and exudations which present themselves in the oral and faucial cavities, in many instances, also aids materially to still further depress the patient, and weaken the reparative processes of his system. These deep-seated abscesses of the neck, when allowed to run their own course, do not exhibit any tendency to a spontaneous cure; but, on the contrary, they always tend to destroy life by burrowing or spreading, etc.; and Dr. Lidell shows that the earlier they are laid open and evacuated the better for both patient and surgeon. As soon as fluctuation is discerned, the abscess cavity should, without delay, be freely laid open, the coagula turned out, the bleeding point, or source of the hemorrhage, brought distinctly into view, and the delinquent vessel itself should be ligatured on each side of the aperture in its walls. But should the ligatures cut through, the actual cautery must be applied to the bleeding point, the primitive carotid artery should be firmly compressed against the cervical vertebræ by the surgeon's thumb or fingers applied on the anterior part of the corresponding side of the neck, between the larynx or trachea and the inner border of the sterno-cleido-mastoid muscle, with force enough to press the artery backward and inward against these vertebræ, and flatten it thereon. Should this procedure fail, it will be advisable, especially in cases where the bleeding proceeds from tonsillary abscesses, to ligature at once the primitive carotid artery.—*Med. Record.*

